# 3D reconstruction based novel methods are more effective than traditional clinical assessment in breast cancer axillary lymph node metastasis prediction

**DOI:** 10.1038/s41598-022-16380-3

**Published:** 2022-07-20

**Authors:** Limeng Qu, Qitong Chen, Na Luo, Piao Zhao, Qiongyan Zou, Xilong Mei, Ziru Liu, Wenjun Yi

**Affiliations:** 1 Department of General Surgery, The Second Xiangya Hospital Of Central South University, No. 139, Renmin Central Road, Changsha, 410011 People’s Republic of China; 2 Department of Orthopaedics, The First Affiliated Hospital of Chongqing Medical University, Chongqing, China; 3 Department of Radiology, The Second Xiangya Hospital of Central South University, Changsha, China; 4 Department of General Surgery, The First People’s Hospital of Changde City, Changde, China

**Keywords:** Breast cancer, Cancer imaging, Cancer models, Metastasis

## Abstract

The status of axillary lymph node metastases determines the treatment and overall survival of breast cancer (BC) patients. Three-dimensional (3D) assessment methods have advantages for spatial localization and are more responsive to morphological changes in lymph nodes than two-dimensional (2D) assessment methods, and we speculate that methods developed using 3D reconstruction systems have high diagnostic efficacy. This exploratory study included 43 patients with histologically confirmed BC diagnosed at Second Xiangya Hospital of Central South University between July 2017 and August 2020, all of whom underwent preoperative CT scans. Patients were divided into a training cohort to train the model and a validation cohort to validate the model. A 3D axillary lymph node atlas was constructed on a 3D reconstruction system to create various methods of assessing lymph node metastases for a comparison of diagnostic efficacy. Receiver operating characteristic (ROC) curve analysis was performed to assess the diagnostic values of these methods. A total of 43 patients (mean [SD] age, 47 [10] years) met the eligibility criteria and completed 3D reconstruction. An axillary lymph node atlas was established, and a correlation between lymph node sphericity and lymph node metastasis was revealed. By continuously fitting the size and characteristics of axillary lymph nodes on the 3D reconstruction system, formulas and models were established to determine the presence or absence of lymph node metastasis, and the 3D method had better sensitivity for axillary lymph node assessment than the 2D method, with a statistically significant difference in the correct classification rate. The combined diagnostic method was superior to a single diagnostic method, with a 92.3% correct classification rate for the 3D method combined with ultrasound. In addition, in patients who received neoadjuvant chemotherapy (NAC), the correct classification rate of the 3D method (72.7%) was significantly higher than that of ultrasound (45.5%) and CT (54.5%). By establishing an axillary lymph node atlas, the sphericity formula and model developed with the 3D reconstruction system achieve a high correct classification rate when combined with ultrasound or CT and can also be applied to patients receiving NAC.

## Introduction

Breast cancer (BC) is the most common malignant tumor and the main cause of female death, and its incidence is still on the rise in China^1^. The clinical axillary lymph node status influences the surgical approach, treatment modality and adjuvant treatment options for patients with BC^2^. Over the decades, the surgical axillary staging and management of early BC has evolved, from complete axillary lymph node dissection (ALND) to sentinel lymph node biopsy (SLNB) to an SLNB-positive waiver of ALND to avoid uncomfortable postoperative drains and the potential for increased morbidity from plasmacytosis, pain, neuropathy, limited arm abduction, lymphedema and an increased risk of cellulitis^3–9^. There is now concern about waiving axillary lymph node biopsy and anterior axillary lymph node biopsy in patients after neoadjuvant chemotherapy (NAC), and there is no consensus on the safety and timing of SLNB in patients receiving NAC. Previous studies have suggested that SLNB may be considered in patients with clinical stage N1 before neoadjuvant therapy and in those with stage N0 after neoadjuvant therapy^10–16^. Therefore, assessment of the axillary lymph node status is very important.

Currently, lymph node palpation, imaging or SLNB is commonly used to assess lymph node metastasis, with a sensitivity of only 32.5–68.3% for determining the presence of lymph node metastasis by palpation. Two-dimensional (2D) imaging methods, such as ultrasound, CT, magnetic resonance imaging (MRI) and positron emission tomography (PET)-CT, are currently the main imaging methods used to diagnose lymph node metastases in BC, with sensitivities of 14–83%^17,18^. The rate of false negatives is 5–10%, and approximately 40–60% of SLN-positive patients have nonsentinel lymph nodes without metastasis; therefore, biopsy of sentinel lymph nodes still has limitations^19,20^. In addition, there is no validated means of accurately distinguishing whether a lymph node is in clinical stage N0, N1 or N2. Therefore, given the diagnostic inefficiency of current assessment methods and the ambiguity of lymph node staging, a validated means of assessing axillary lymph nodes is needed.

The ratio of the long axis to the short axis of metastatic lymph nodes is reduced, and the cortex is abnormally thickened, which results in the metastatic lymph nodes tending to be spherical in shape with the normal lymph nodes remaining oval^21^. Current 2D assessment methods do not accurately show the spherical shape of the suspected lymph node, and we can analyze the lesion only by looking at the scan and imagining its three-dimensional (3D) shape. The diagnosis relies mainly on the subjective judgment and experience of the physician. We speculate that there is significant interest in using morphological changes in lymph node sphericity to assess the status of lymph nodes. In our previous study, we reported that 3D reconstruction systems are more likely to detect more suspicious areas of metastasis than CT or ultrasound, which greatly facilitates accurate assessment of the lesion area and facilitates the associated surgery. We therefore aimed to determine whether the use of a 3D reconstruction system to develop various diagnostic methods at a 3D level might improve the diagnostic efficacy of the assessment methods; therefore, we enrolled eligible patients and performed 3D reconstruction on CT images from these patients using different methods to assess the lymph nodes to examine the difference in diagnostic efficacy between the different methods.

## Methods

### Patient selection

**Figure 1:**
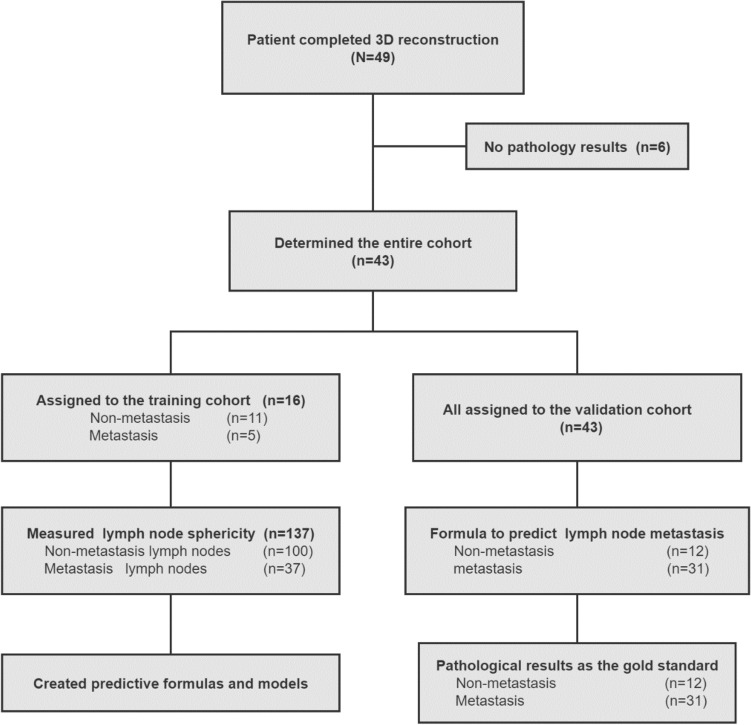
Flow chart of the exploratory study.

Between July 2017 and August 2020, we enrolled 49 patients with early-stage BC. 3D reconstruction of CT images of the lung was completed in all of these patients preoperatively with the 3D reconstruction system, but six patients were excluded because they did not undergo surgery or biopsy and lacked pathological results to determine the presence of metastases in the axillary lymph nodes, resulting in a total of 43 patients being included in this study (Fig. 1). The clinicopathological characteristics of patients in the entire cohort are presented in Table 1. All 43 patients were female, aged 30 to 74 years, and the median age was 47 years. Patients whose lymph nodes were all metastatic (*n* = 5) and patients whose lymph nodes were not metastatic (*n* = 11) were divided into a training cohort to train the model. According to the pathological examination, there were 137 lymph nodes in the training cohort, of which 37 lymph nodes had metastasis and 100 lymph nodes did not have metastasis, and all patients were included in the validation set to validate the model. All patients had lymph node pathological examination results corresponding to 3D reconstruction system images. The study was reviewed and approved by the Research Ethics Committee of the Second Xiangya Hospital of Central South University and conducted in accordance with the ethical guidelines of the Declaration of Helsinki, and obtained informed consent for the published identification images and information.

**Table 1:** Clinicopathological characteristics of patients in the entire cohort.

	No metastasis	Metastasis	Total	*P*-Value
	*N* (%)	*N* (%)	*N* (%)	
Age, mean (SD), y	46 (9)	48 (11)	47 (10)	0.542
Tumor size, median (IQR), cm	2.3 (1.9–2.8)	3.0 (2.4–3.3)	3.0 (2.0–3.0)	0.011
**Histological grade**				
I	3 (25.00)	1 (3.23)	4 (9.30)	0.0523
II	6 (50.00)	25 (80.65)	31 (72.09)	
III	3 (25.00)	5 (16.13)	8 (18.60)	
**Pathological T stage**				
1	6 (50.00)	7 (22.58)	13 (30.23)	0.1729
2	6 (50.00)	22 (70.97)	28 (65.12)	
3	0 (0.00)	2 (6.45)	2 (4.65)	
**Pathological N stage**				
0	12 (100.00)	0 (0.00)	12 (27.91)	< 0.0001
1	0 (0.00)	16 (51.61)	16 (37.21)	
2	0 (0.00)	7 (22.58)	7 (16.28)	
3	0 (0.00)	8 (25.81)	8 (18.60)	
**Molecular subtype**				
Luminal A	2 (16.67)	7 (22.58)	9 (20.93)	0.8696
Luminal B	2 (16.67)	7 (22.58)	9 (20.93)	
ERBB2-positive	5 (41.67)	9 (29.03)	14 (32.56)	
Triple negative	3 (25.00)	8 (25.81)	11 (25.58)	
**ER**				
Negative	5 (41.67)	13 (41.94)	18 (41.86)	1
Positive	7 (58.33)	18 (58.06)	25 (58.14)	
**PR**				
Negative	8 (66.67)	13 (41.94)	21 (48.84)	0.2648
Positive	4 (33.33)	18 (58.06)	22 (51.16)	
**HER2**				
Negative	7 (58.33)	22 (70.97)	29 (67.44)	0.667
Positive	5 (41.67)	9 (29.03)	14 (32.56)	
**Ki-67**				
≤ 20	3 (25.00)	8 (25.81)	11 (25.58)	1
> 20	9 (75.00)	23 (74.19)	32 (74.42)	
**Neoadjuvant**				
Yes	3 (25.00)	16 (51.61)	19 (44.19)	0.2172
No	9 (75.00)	15 (48.39)	24 (55.81)	
Total	12	31	43	

### 3D reconstruction system

The 3D reconstruction system adopted the Intco system and Shanghai Evision Medical Technology Co, Vitaworks system(http://www.vitaworks.cn/) andthe 3D reconstruction images of each patient can be viewed through third party software (VitaWorks, China).

### Clinicopathological features

We recorded clinicopathological features, including the number of lymph nodes, estrogen receptor (ER) status, progesterone receptor (PR) status, human epidermal growth factor receptor 2 (HER2) status, and Ki-67 index, according to the pathology reports from the Department of Pathology of the Second Xiangya Hospital of Central South University. Core biopsy samples and surgical specimens were evaluated using standard hematoxylin and eosin staining, immunohistochemistry (IHC), and fluorescence or chromogenic in situ hybridization (FISH) (or both) to determine the histological subtype, Ki-67 index, and ER, PR, and HER2 statuses^22^. Cutoff values for ER, PR, and Ki-67 were 1%, 1%, and 20%, respectively^23^. The cutoff value for HER2 was set at 3 + for IHC and 2.0 for FISH^24^. Any cases of HER2 found to be 2 + on IHC were examined by FISH and classified as HER2-positive if the HER2 gene was found to be amplified. BCs were classified as the luminal A, luminal B, HER2 overexpression or triple negative (TN) subtype. The clinicopathological characteristics of all patients are summarized in Table 1.

### Diagnostic criteria of the 3D reconstruction system

BC patients whose pathological examination showed that the axillary lymph nodes were all metastatic or all non-metastatic, and they were categorized as metastatic and non-metastatic patients, then the corresponding lymph node images in the 3D reconstruction system should be represented as metastatic or non-metastatic. BC patients whose pathological examination showed partial metastasis of axillary lymph nodes, who had part of their axillary lymph nodes as metastatic and part as non-metastatic lymph nodes, were considered as patients with lymph node metastasis as a whole, and this group of patients was categorized as metastatic in the entire cohort and the validation cohort.

The sphericity of a lymph node in the 3D reconstruction system was also measured (Fig. S1).

### Statistical analysis

Continuous variables were compared using an independent t test, and 2-group categorical variables were compared using a chi-square test (χ2 test). The comparison of correct classification rates between the two diagnostic methods was performed using the χ2 test with four-compartment table information. The prognostic or predictive accuracy of the 2D formula $\frac{a}{c}$ and the sphericity formula $\frac{{2 \times \sqrt[3]{{\frac{{abc}}{8}}}}}{c}$ was assessed using receiver operating characteristic (ROC) curve analysis and by calculating the area under the curve (AUC). Statistical analyses and the creations of decision tree models and random forest models were performed with SPSS statistical software v25.0 and R version 4.0.3 (R Project for Statistical Computing) of R studio^25^. ROC curve analysis with the maximum Youden index was used to obtain the optimal cutoff value for each continuous variable. A *p* value < 0.05 was determined to be statistically significant.

### Ethics approval and consent to participate

The study was reviewed and approved by the Research Ethics Committee of the Second Xiangya Hospital of Central South University and conducted in accordance with the ethical guidelines of the Declaration of Helsinki, and obtained informed consent for the published identification images and information. Informed consent was obtained from each participation for participating in the study.

## Results

### Construction of the axillary lymph node atlas of BC

**Figure 2:**
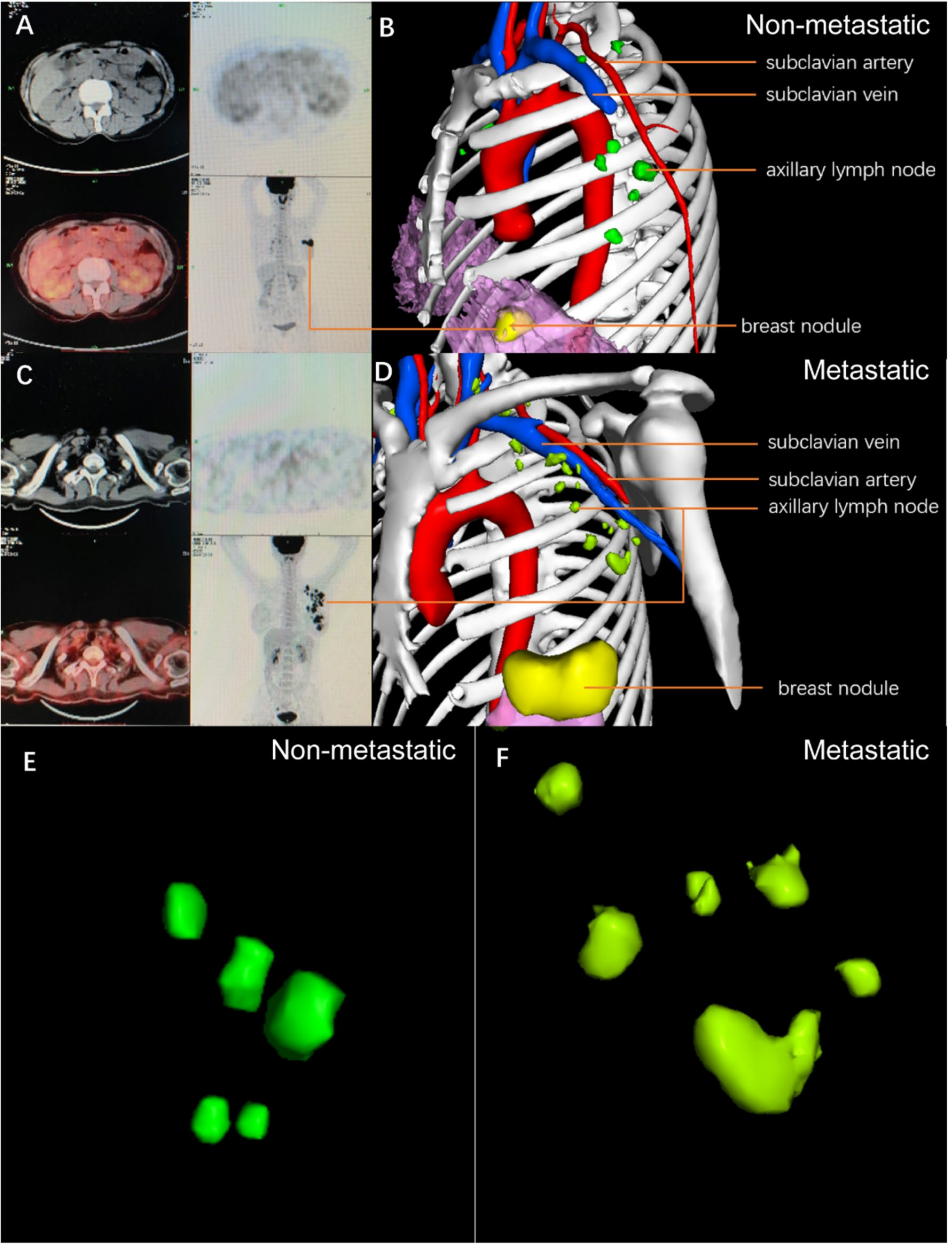
Comparison of PET-CT images with the 3D reconstruction system for the display of suspected metastatic lymph nodes. (**A**) and (**C**): Positron emission tomography/computed tomography images of non-metastatic (**A**) and metastatic patients (**C**); (**B**) and (**D**): 3D reconstruction images of non-metastatic (**B**) and metastatic patients (**D**); (**E**) and (**F**): 3D reconstruction local magnified images of non-metastatic (**E**) and metastatic patients (**F**).

Using 3D-reconstructed images from 43 patients, we constructed a new axillary lymph node atlas. In some patients who underwent PET-CT, we compared the PET-CT and 3D reconstruction images from two patients: one patient had axillary lymph node metastasis, and the other had no metastasis (Fig. 2). In patients without lymph node metastasis, the shape of the lymph nodes was regular, and most of the lymph nodes were ellipsoidal (Fig. 2A, B, E). In patients with lymph node metastasis, the shape of the lymph nodes began to become irregular, and most lymph nodes changed from oval to more spherical (Fig. 2C, D, F).

### Formulas and models for predicting lymph node metastasis in the training cohort

**Figure 3:**
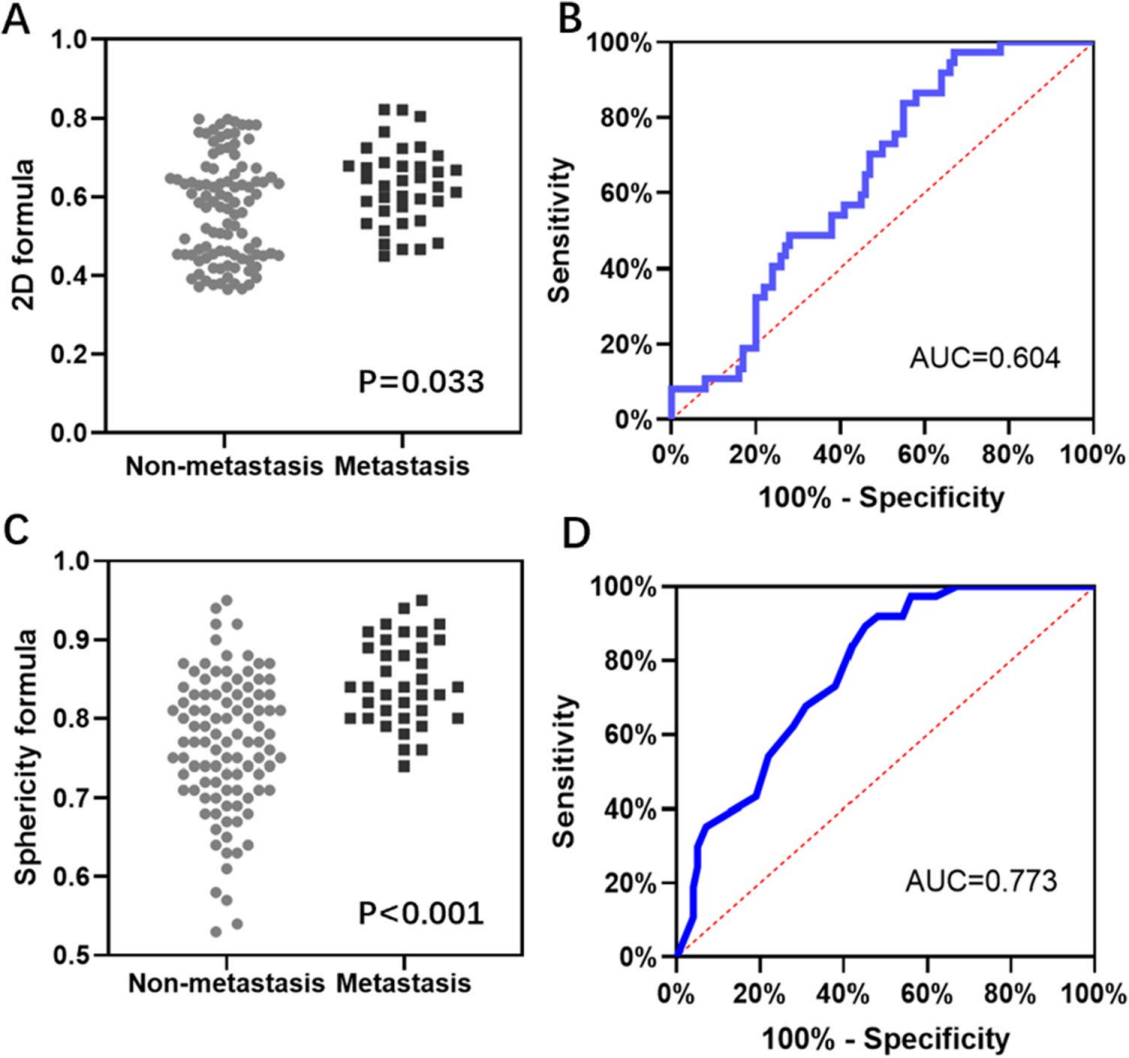
Evaluation of the diagnostic efficacy of the 2D formula and the sphericity formula. (**A**) and (**C**): Visualization of the values measured in 137 lymph nodes using the 2D formula(**A**) and 3D formula(**C**); (**B**) and (**D**): ROC curves for the diagnosis of lymph node metastases by the 2D formula (**B**) and the sphericity formula (**D**).

**Figure 4:**
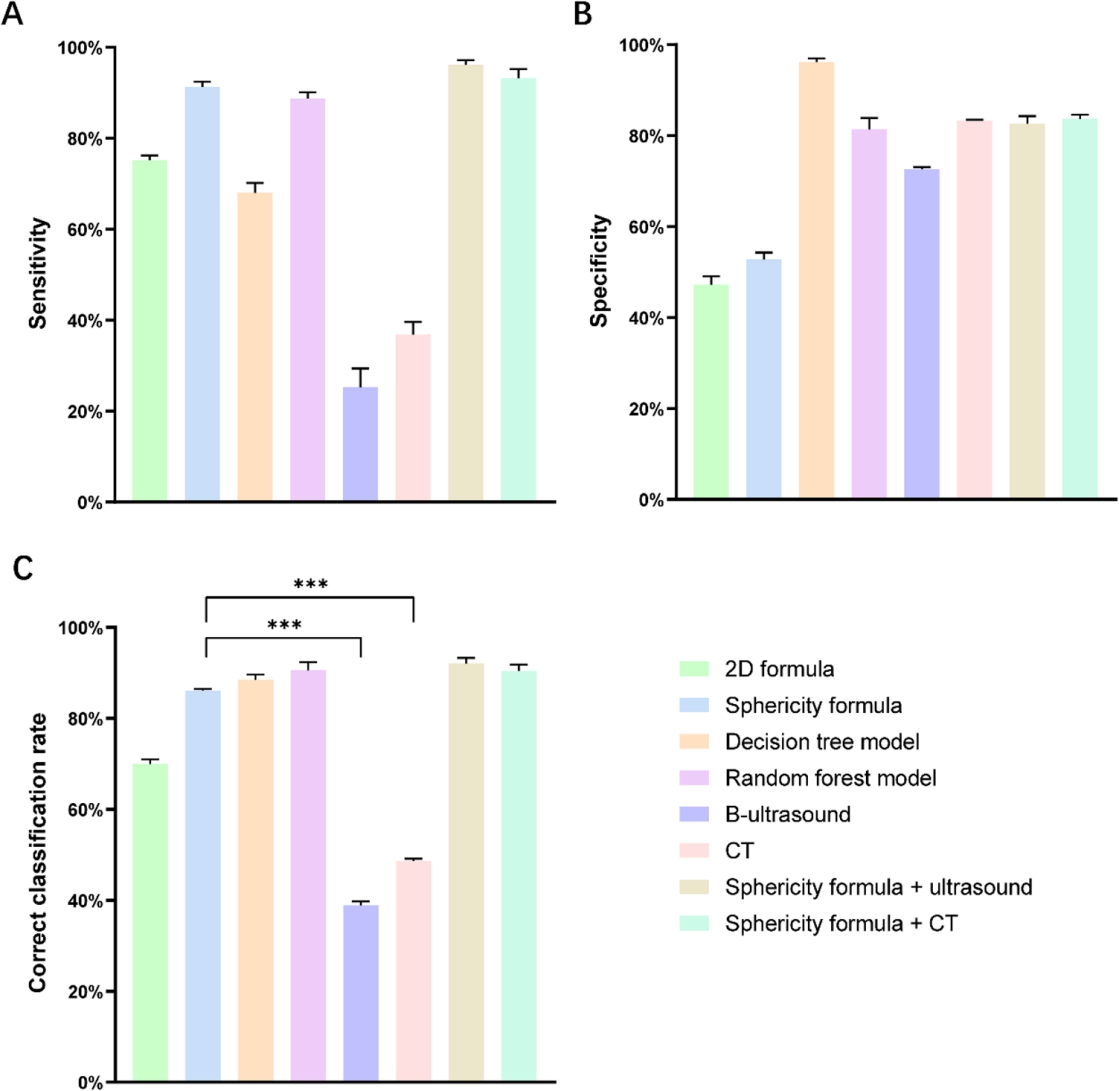
Comparison of the diagnostic efficacy of lymph node assessment methods. (**A**) and (**B**): Comparison of the sensitivity (**A**) and specificity (**B**) of various assessment methods for the diagnosis of lymph node metastases; (**C**): Comparison of correct classification rates for lymph node assessment methods. ***p < 0.0001 by the χ2 test.

After determining that the shape of the metastatic lymph nodes may be more spherical than oval, we suspected that the difference in sphericity between the lymph nodes may help us to identify diseased lymph nodes. In a training cohort containing 16 patients, we measured the sphericity of a total of 137 lymph nodes, 37 of which had metastases on histopathological examination and 100 of which did not. Using the variation in sphericity of the lymph nodes, we created a sphericity formula. The ROC curve for this formula was plotted by substituting the a, b and c values into the sphericity formula, with an AUC of 0.773 (Fig. 3D), a sensitivity of 91.9% and a specificity of 52.0% (Fig. 4A, B, Table S1), with significantly higher values in the metastatic group than in the nonmetastatic group (Fig. 3C). We also used a 2D plane-based formula, which uses the ratio of the short diameter to the long diameter to identify metastatic lymph nodes, with an AUC of 0.604 (Fig. 3B), a sensitivity of 75.5% and a specificity of 46.3% (Fig. 4A, B, Table S1), all of which were lower than those obtained from the sphericity formula, with no significant difference between the metastatic and nonmetastatic groups (Fig. 3A). Thus, the diagnostic performance of the 3D formula was significantly better than that of the 2D formula.

To further improve diagnostic efficacy, we attempted predictive classification using a decision tree model. As shown in Fig. S2, the 137 lymph node samples from the training set were divided into nodes according to the values of the independent variables a, b and c so that the distribution of the dependent variable within the terminal nodes converged as closely as possible. The model had a total of 19 prediction errors, of which 16 were misdiagnosed and three were missed, with an accuracy of 86.1%, a sensitivity of 68.0% and a specificity of 96.6% (Table S1). We also ranked the importance of the three predictor variables, with the target variable c being the most important, followed by a (Fig. S3A).

On the basis of the decision tree model, we used an algorithm called random forest to establish a random forest model to further improve prediction efficiency. At present, the random forest algorithm has superior classification performance. The random forest prediction model had an AUC value of 0.844, a sensitivity of 88.9% and a specificity of 80.0% (Fig. S3B). It had the lowest error rate when there were 100 decision trees, and the prediction variable a became more important in this model (Fig. S3C, D). Among the 137 samples, there were only 5 prediction errors, the accuracy was as high as 96.3%, and the performance was greatly improved compared with that of the decision tree model.

Therefore, in this study, four methods, 2D formula, sphericity formula, decision tree model and random forest model, were used to predict and classify 137 lymph nodes in the training cohort, and formulas and models were established to determine the presence or absence of lymph nodes.

### Overall validation in the validation cohort

This study provides an overall validation of the methods of each study. In the validation cohort, the sphericity formula had a much higher correct classification rate of 86% compared that of the 2D formula (69.8%). In the decision tree model and random forest model, the correct classification rate improved to 88.4% and 90.7%, respectively, which were significantly different compared to those of the 2D formula (*p* = 0.031; *p* = 0.014, Table S3).

Next, to further assess the performance of each method, we compared each diagnostic method with ultrasound. A total of 39 patients underwent preoperative 3D reconstruction and ultrasound. Ultrasound showed no metastases in 7 patients, lymph node enlargement in 15 patients, lymph node supraphysiology in 6 patients, metastatic lymph nodes in 10 patients and heterogeneous lymph nodes in 1 patient. Compared with the pathological findings, ultrasound correctly diagnosed 7 cases of metastatic lymph nodes and 8 cases of nonmetastatic lymph nodes; ultrasound missed the diagnosis in 21 patients and misdiagnosed 3 patients, with a correct classification rate of only 38.5%, which was significantly lower than that of each diagnostic method (Fig. 4C, Tables S2-3).

Finally, we attempted an evaluation with CT. The correct classification rate was 48.8%, and this rate was significantly different from those of the other diagnostic methods (Fig. 4C, Tables S2-3).

### Combined diagnostic method is better than a single diagnostic method

After assessing the diagnostic efficacy of each method, we speculated whether the combination of two methods would further improve diagnostic accuracy; here, we combined the sphericity formula with ultrasound and CT. We found that the sphericity formula had a sensitivity of 96.4% and a specificity of 81.8% when combined with ultrasound for diagnosis. When combined with CT, it had a sensitivity of 93.5% and a specificity of 83.3%, with high diagnostic efficacy (Fig. 4A, B). Upon validation in 39 patients, ultrasound missed the diagnosis in one patient and misdiagnosed two patients, with a correct classification rate of 92.3%; in the total validation set, ultrasound missed the diagnosis in 2 patients and misdiagnosed 2 patients, with a correct classification rate of 90.7% when the sphericity formula was combined with CT (Fig. 4C, Table S2). This result suggests that diagnostic efficacy is improved when diagnostic methods are combined.

### Assessment of lymph nodes after NAC

**Figure 5:**
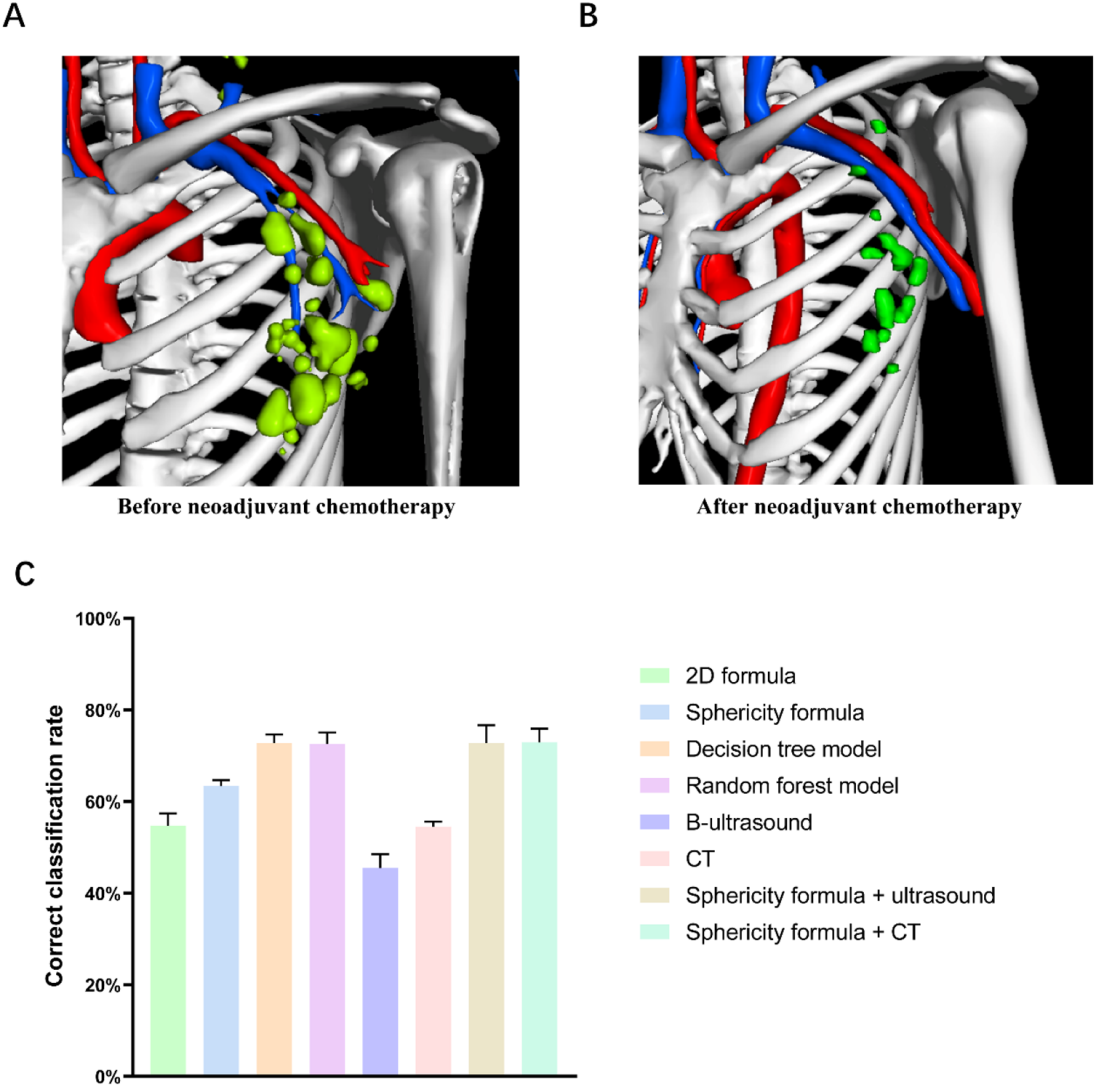
3D-reconstructed images before and after neoadjuvant chemotherapy and comparison of the assessment efficacy of each method in patients receiving neoadjuvant chemotherapy. (**A**) Before neoadjuvant chemotherapy; (**B**) After neoadjuvant chemotherapy; (**C**) Comparison of correct classification rates of lymph node assessment methods in patients receiving neoadjuvant chemotherapy.

Considering that the assessment of axillary lymph nodes after NAC is also an important part of subsequent treatment decisions, we assessed lymph nodes in patients after NAC using the methods described above. We included a total of 11 patients, five of whom had lymph node metastases and six of whom did not, as determined by the pathological findings post neoadjuvant surgery. These patients underwent 3D CT reconstruction before and after NAC. The morphology of the lymph nodes changed significantly before and after NAC (Fig. 5A, B). We attempted to measure the sphericity of the patients' lymph nodes after NAC in the 3D reconstruction system, and a total of 90 lymph nodes were measured. Among the various assessment methods, ultrasound had a low correct classification rate of 45.5%, followed by the 2D formula and CT (54.5%); the sphericity formula had a better correct classification rate than the 2D method (63.6%); and the decision tree and random forest models and the combined diagnostic methods had the highest correct classification rate (72.7%) (Fig. 5C, Table S4-5). Overall, the diagnostic efficacy of the 3D method and the combined diagnostic method was better than that of the 2D method, but the diagnostic efficacy decreased in patients not receiving NAC.

## Discussion

A clinical axillary lymph node evaluation is an important part of the diagnosis and treatment of BC^26–28^. According to the number and location of axillary lymph node metastases, BC lymph node staging is usually divided into N0, N1, N2, and N3. The higher the stage is, the worse the prognosis^29^. In the past, routine axillary lymph node dissection in BC surgery had no prognostic value for patients in stage N0 but produced a series of complications, affecting their quality of life^30,31^. In the 2010s, the American Association of Surgeons Oncology Group (ACOSOG) Z0011 trial changed the clinical model of axillary treatment, for BC patients with cN0 disease, when SLNB instead of ALND became a possibility^32–34^. Some studies that have also demonstrated comparable disease-free survival (DFS) between SLNB and ALND in patients with early-stage BC and only 1–2 sentinel lymph nodes with micrometastases^20,35,36^. The feasibility of SLNB in patients with axillary lymph node-negative BC after NAC is still under study^14,37–41^.

Although SLNB provides a solid foundation for the precise treatment of BC, with advances in medical technology, the exemption of SLNB and precision axillary surgery has become a current concern. Tailored axillary surgery with or without ALND followed by radiation therapy (TAXIS), as an emerging surgical modality for patients with clinically node-positive BC, evaluates survival after selectively removing positive lymph nodes as a less extensive surgical option^42^; this finding suggests that axillary surgery is becoming increasingly accurate and noninvasive. However, there is a need for more accurate preoperative diagnosis and imaging methods to predict axillary lymph node metastases.

In patients with positive clinical axillary lymph nodes that turn negative after NAC, there is still no conclusive evidence to confirm the reliability of SLNB after NAC. On the one hand, there are difficulties associated with determining the spatial localization of lymph nodes in stage cN1; on the other hand, there is still a certain rate of false negatives, and the accuracy of the assessment is poor.

Current non-invasive staging of axillary imaging methods cannot replace invasive staging in pathology, and the accurate clinical diagnosis of axillary lymph nodes often requires an invasive assessment method^26^. As the trend toward less invasive axillary surgery continues, imaging may need to play a more precise but comprehensive role in the evaluation of axillary lymph nodes. We need to develop a new imaging technique to provide a more accurate assessment of preoperative and post-treatment response, and in the future exempt a proportion of patients from invasive assessment.

As a new field of multidisciplinary research, 3D reconstruction of medical images has facilitated the development of medicine through the combination of computer graphics and image processing techniques and has provided a new way of thinking about the assessment of lymph nodes^43,44^. In this study, a 3D reconstruction system was used to create a new axillary lymph node atlas for the diagnostic assessment of lymph node metastases to determine the surgical approach, assist in follow-up treatment and improve patient prognosis; this was also its first application in axillary lymph nodes.

In our study, we exploratively analyzed the lymph nodes of 43 patients diagnosed with BC who had completed 3D reconstruction and found a correlation between lymph node sphericity and lymph node metastasis. By continuously fitting the size and characteristics of axillary lymph nodes on the 3D reconstruction system, we developed a formula and model for determining the presence of lymph node metastasis, and the 3D formula had a higher AUC and accuracy for the detection of axillary metastatic lymph nodes than the 2D formula. In addition, the decision tree model and random forest model we established further improved diagnostic efficacy, and both achieved high correct classification rates in the validation cohort, indicating that the application of artificial intelligence to medical diagnoses will open the door to precise axillary surgery. Compared with traditional imaging methods such as ultrasound and CT, the 3D sphericity formula and model are more effective in the diagnosis of metastatic lymph nodes, indicating that the 3D method can spatially improve the diagnostic effectiveness and facilitate the spatial localization of lymph nodes. In addition, we used the sphericity formula in combination with ultrasound or CT and found that the combined diagnostic method was superior to a single diagnostic method. Finally, we conducted a preliminary study on the assessment of axillary lymph nodes in patients receiving NAC. Diagnostic efficacy was reduced using each of the diagnostic methods but was still higher than that of ultrasound and CT, possibly due to some changes in the size and shape of the lymph nodes in patients receiving NAC.

Although we generated a novel formula and model for diagnosing lymph node metastasis in BC, there are still some limitations. First,the number of patients in this study was small due to our short time of using the 3D reconstruction system. The established decision tree model and random forest model may have the risk of overfitting, and more patients need to be included in our study in the future. Secondly, these models lacked external validation, and subsequent external validation is needed to improve the confidence of the results, and finally inevitably, there may have been some manual measurement errors, which cannot be completely avoided.

In conclusion, in this study, we constructed a novel BC axillary lymph node atlas, created the best diagnostic formula and model reflecting the morphological changes in metastatic lymph nodes for lymph node assessment, thus distinguishing metastatic lymph nodes from nonmetastatic lymph nodes with high sensitivity and specificity, and provided new insights and a method for the spatial localization and combined diagnosis of axillary lymph nodes in BC. The combination of 3D methods with traditional diagnostic methods and molecular diagnostics has potential clinical application prospects that need to be further validated in studies on large samples.

## Supplementary Information

Supplementary Information.

## Data Availability

All remaining data are available within the article or available from the authors upon request.

## Abbreviations

BC: Breast cancer
3D: Three-dimensional
2D: Two-dimensional
ROC: Receiver operating characteristic
NAC: Neoadjuvant chemotherapy
ALND: Axillary lymph node dissection
SLNB: Sentinel lymph node biopsy
MRI: Magnetic resonance imaging,
PET: Positron emission tomography
ER: Estrogen receptor
PR: Progesterone receptor
HER2: Human epidermal growth factor receptor 2
IHC: Immunohistochemistry
FISH: Fluorescence or chromogenic in situ hybridization
TN: Triple negative
ROC: Receiver operating characteristic
AUC: The area under the curve
ACOSOG: The American Association of Surgeons Oncology Group
DFS: Disease-free survival
TAXIS: Tailored axillary surgery with or without ALND followed by radiation therapy
