# Supplementary material for: 3D reconstruction based novel methods are more effective than traditional clinical assessment in breast cancer axillary lymph node metastasis prediction
Source: Sci Rep. 2022 Jul 20;12:12425. doi: 10.1038/s41598-022-16380-3 (PMC9300607; doi:10.1038/s41598-022-16380-3)
Supplement: Supplementary file 1 — Supplementary Information. [file 41598_2022_16380_MOESM1_ESM.docx]

**3D reconstruction based novel methods are more effective than traditional clinical assessment in breast cancer axillary lymph node metastasis prediction**

**Authors and affiliations**

Limeng Qu^1#^, Qitong Chen^1^, Na Luo^1,4^, Piao Zhao^2^, Qiongyan Zou^1^, Xilong Mei^3^, Ziru Liu^1*^, Wenjun Yi^1*^

1. Department of General Surgery, The Second Xiangya Hospital Of Central South University, Changsha, China.

2. Department of Orthopaedics, The First Affiliated Hospital of Chongqing Medical University, Chongqing, China.

3. Department of Radiology, The Second Xiangya Hospital Of Central South University, Changsha, China.

4. Department of General Surgery, The First People’s Hospital of Changde City, Changde, China.

**^*^Corresponding author:**

Dr. Ziru Liu: Department of General Surgery, The Second Xiangya Hospital of Central South University, No. 139, Renmin Central Road, Changsha, 410011, P.R. China, +8613467511214, [liuziru@csu.edu.cn](mailto:liuziru@csu.edu.cn)

Prof. Wenjun Yi, Department of General Surgery, The Second Xiangya Hospital of Central South University, No. 139, Renmin Central Road, Changsha, 410011, P.R. China, +8618608403318, yiwenjun@csu.edu.cn

**^#^First author: Limeng Qu**

Mailing address: No.139 Middle Renmin Road, Changsha, Hunan 410011, P.R. China

Telephone number: +86-13973650340,

E-mail address: 208212267@csu.edu.cn

**Conflict of Interest Statement:** None.

**Keywords:** breast cancer; axillary lymph node metastasis; three-dimensional reconstruction; diagnosis method

**Supplementary Materials**


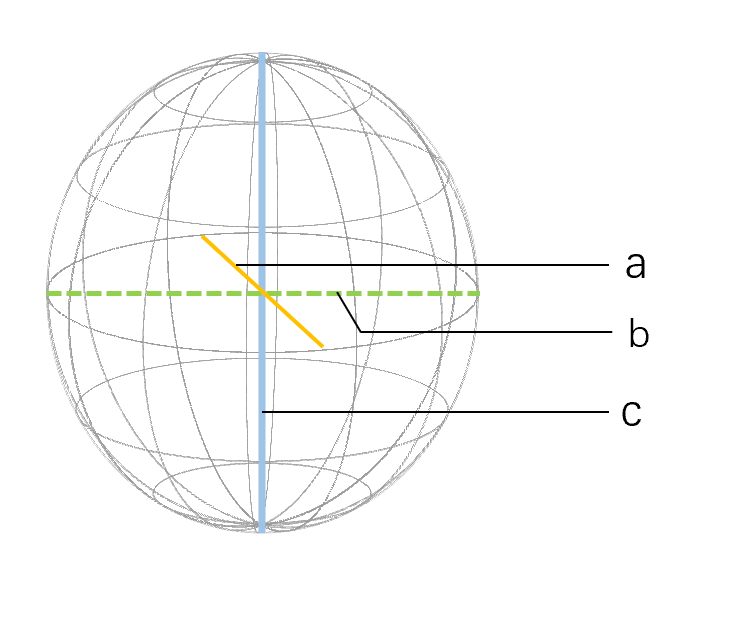


Figure S1 Sphericity measurement diagram, the lymph node is approximated as an ellipsoid, c is the longest diameter of the lymph node, a is the shortest diameter passing through the midpoint of c and perpendicular to c, and b is the longest diameter of the plane


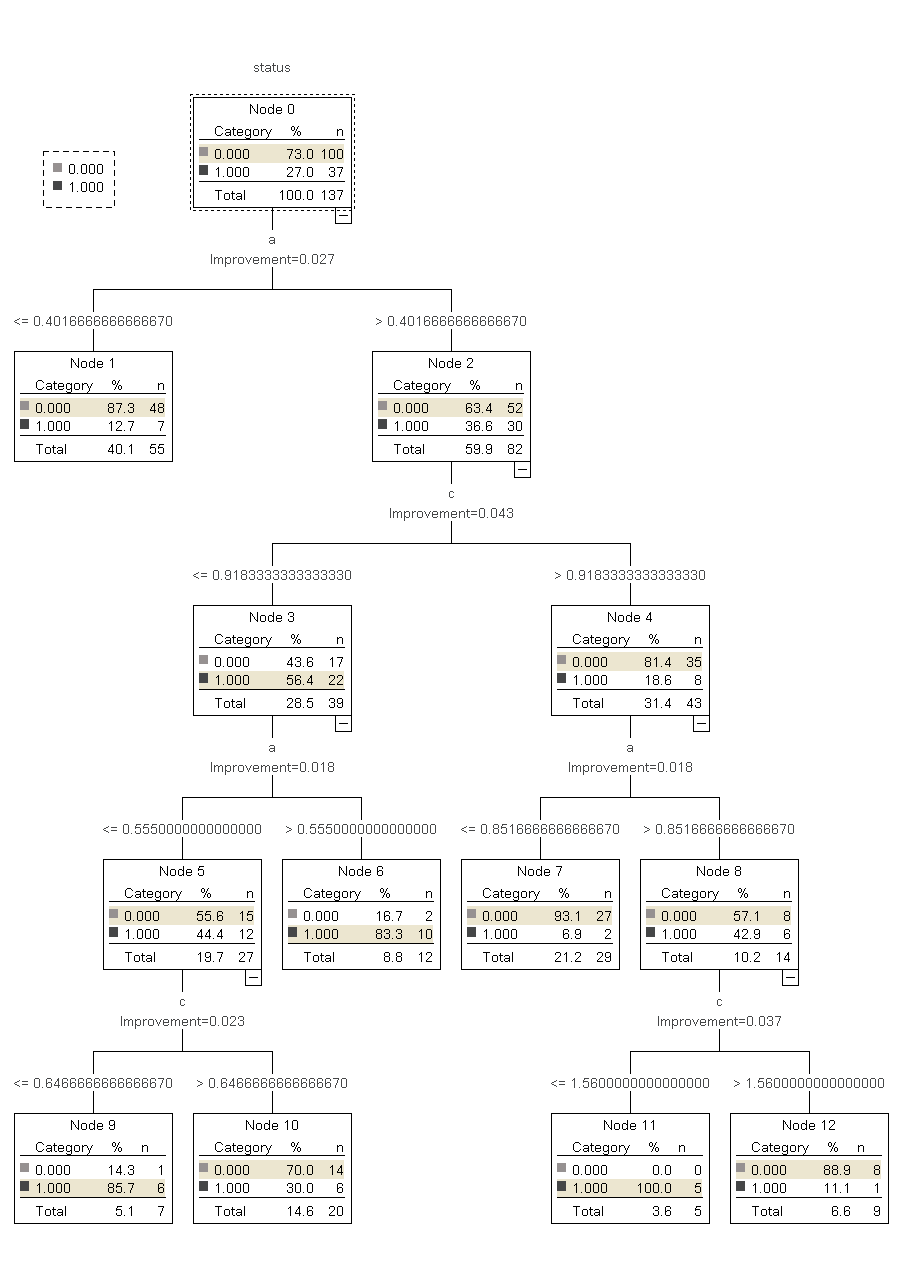


Figure S2. Decision tree model. Dividing the sample by nodes according to the value of the independent variable, the sample size of all terminals was equal to the root knot sample size. 0 indicates no lymph node metastasis, and 1 indicates lymph node metastasis.


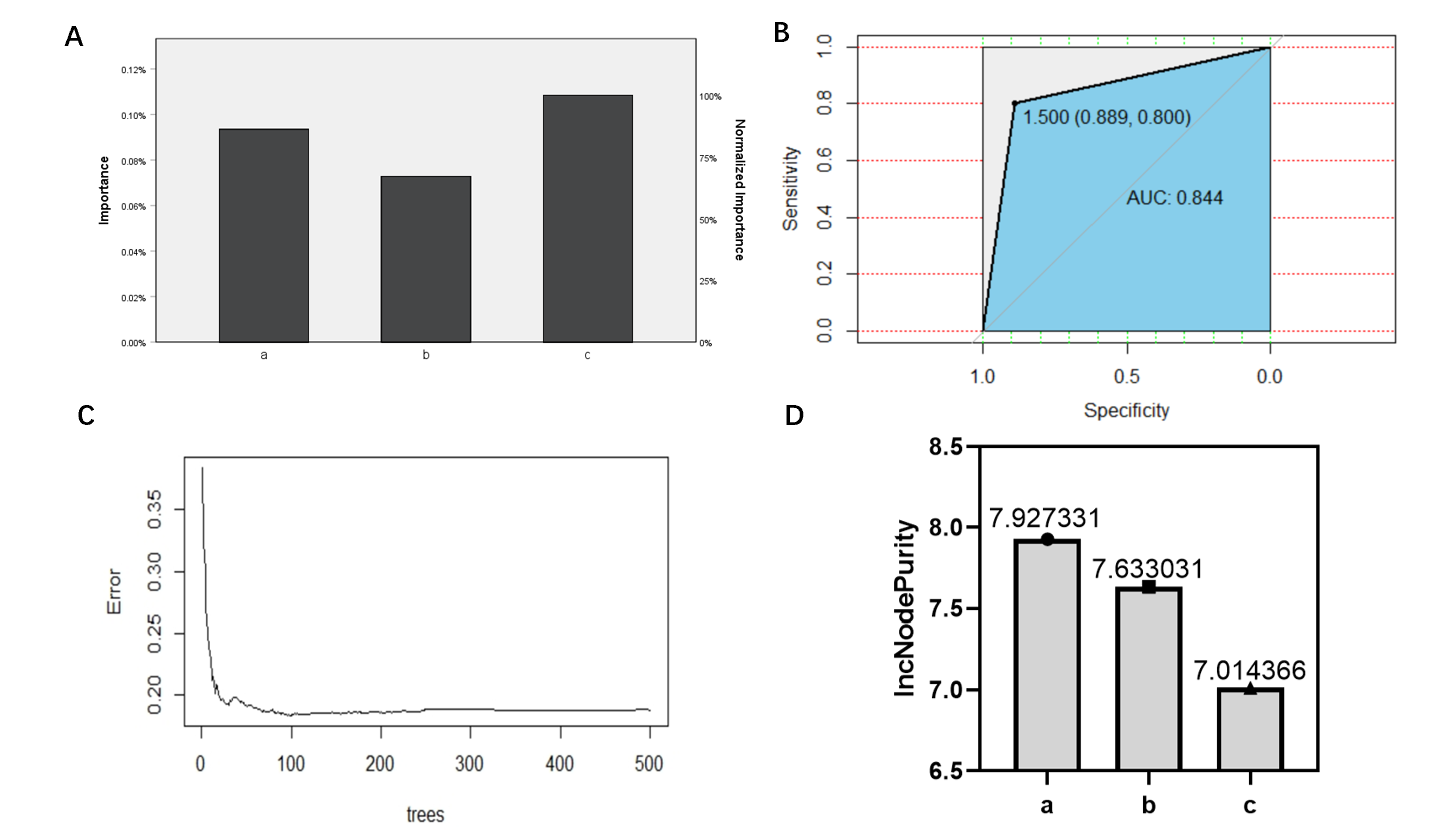


Figure S3. Importance of variables in decision tree models and performance evaluation of random forest models

Table S1 Evaluation Index of assessment methods for diagnosis of lymph node metastasis by 3D Reconstruction system and traditional imaging

|  | Sensitivity | Specificity | False positive rate | False negative rate |
| --- | --- | --- | --- | --- |
| 2D formula | 75.5% | 46.3% | 53.7% | 25.5% |
| Sphericity formula 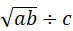 | 91.9% | 52.0% | 48.0% | 8.1% |
| Decision tree model | 68.0% | 96.6% | 3.4% | 32.0% |
| Random forest model | 88.9% | 80.0% | 20.0% | 11.1% |
| B-ultrasound | 25.0% | 72.7% | 27.3% | 75.0% |
| CT | 35.5% | 83.3% | 16.7% | 64.5% |
| Sphericity formula combined with ultrasound | 96.4% | 81.8% | 18.2% | 3.6% |
| Sphericity formula combined with CT | 93.5% | 83.3% | 16.7% | 6.5% |

Table S2 Evaluation of the validity of the assessment methods

|  |  | Pathological examination results | | |  |
| --- | --- | --- | --- | --- | --- |
|  |  | Metastasis | Non-metastasis | Total | Correct classification rate |
| **2D formula** | Metastasis | 24 | 6 | 30 | 69.8% |
|  | Non-metastasis | 7 | 6 | 13 |  |
|  | Total | 31 | 12 | 43 |  |
| **Sphericity formula** | Metastasis 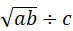 | 28 | 3 | 31 | 86.0% |
|  | Non-metastasis | 3 | 9 | 12 |  |
|  | Total | 31 | 12 | 43 |  |
| **Decision tree model** | Metastasis | 28 | 2 | 30 | 88.4% |
|  | Non-metastasis | 3 | 10 | 13 |  |
|  | Total | 31 | 12 | 43 |  |
| **Random forest model** | Metastasis | 29 | 2 | 31 | 90.7% |
|  | Non-metastasis | 2 | 10 | 12 |  |
|  | Total | 31 | 12 | 43 |  |
|  | Metastasis | 7 | 3 | 10 | 38.5% |
| **B-ultrasound** | Non-metastasis | 21 | 8 | 29 |  |
|  | Total | 28 | 11 | 39 |  |
| **CT** | Metastasis | 11 | 2 | 13 | 48.8% |
|  | Non-metastasis | 20 | 10 | 30 |  |
|  | Total | 31 | 12 | 43 |  |
| **Sphericity formula combined with ultrasound** | Metastasis | 27 | 2 | 29 | 92.3% |
|  | Non-metastasis | 1 | 9 | 10 |  |
|  | Total | 28 | 11 | 39 |  |
| **Sphericity formula combined with CT** | Metastasis | 29 | 2 | 31 | 90.7% |
|  | Non-metastasis | 2 | 10 | 12 |  |
|  | Total | 31 | 12 | 43 |  |

Table S3 Statistical variability analysis for each of the two methods in the validation cohort

|  | 2D formula | Sphericity formula | Decision tree model | Random forest model | B-ultrasound | CT | Sphericity formula combined with ultrasound | Sphericity formula combined with CT |
| --- | --- | --- | --- | --- | --- | --- | --- | --- |
| 2D formula | - | 0.059 | **0.031** | **0.014** | **0.004** | **0.039** | **0.01** | **0.014** |
| Sphericity formula 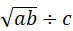 | 0.059 | - | 0.5 | 0.369 | 0.000 | 0.000 | 0.293 | 0.369 |
| Decision tree model | **0.031** | 0.5 | - | 0.5 | **0.000** | **0.000** | 0.413 | 0.5 |
| Random forest model | **0.014** | 0.369 | 0.5 | - | **0.000** | **0.000** | 0.555 | 0.644 |
| B-ultrasound | **0.004** | **0.000** | **0.000** | **0.000** | - | 0.235 | **0.000** | **0.000** |
| CT | **0.039** | **0.000** | **0.000** | **0.000** | 0.235 | - | **0.000** | **0.000** |
| Sphericity formula combined with ultrasound | **0.01** | 0.293 | 0.413 | 0.555 | **0.000** | **0.000** | - | 0.555 |
| Sphericity formula combined with CT | **0.014** | 0.369 | 0.5 | 0.644 | **0.000** | **0.000** | 0.555 | - |

A p value<0.05 was determined to be statistically significant.

Table S4 Evaluation of the validity of lymph node assessment methods in post-neoadjuvant patients

|  |  | Pathological examination results | | |  |
| --- | --- | --- | --- | --- | --- |
|  |  | Metastasis | Non-metastasis | Total | Correct classification rate |
| **2D formula** | Metastasis | 3 | 3 | 6 | 54.5% |
|  | Non-metastasis | 2 | 3 | 5 |  |
|  | Total | 5 | 6 | 11 |  |
| **Sphericity formula** | Metastasis 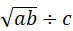 | 4 | 3 | 7 | 63.6% |
|  | Non-metastasis | 1 | 3 | 4 |  |
|  | Total | 5 | 6 | 11 |  |
| **Decision tree model** | Metastasis | 3 | 1 | 8 | 72.7% |
|  | Non-metastasis | 2 | 5 | 3 |  |
|  | Total | 5 | 6 | 11 |  |
| **Random forest model** | Metastasis | 4 | 2 | 6 | 72.7% |
|  | Non-metastasis | 1 | 4 | 5 |  |
|  | Total | 5 | 6 | 11 |  |
|  | Metastasis | 2 | 3 | 5 | 45.5% |
| **B-ultrasound** | Non-metastasis | 3 | 3 | 6 |  |
|  | Total | 5 | 6 | 11 |  |
| **CT** | Metastasis | 2 | 2 | 4 | 54.5% |
|  | Non-metastasis | 3 | 4 | 7 |  |
|  | Total | 5 | 6 | 11 |  |
| **Sphericity formula combined with ultrasound** | Metastasis | 4 | 2 | 6 | 72.7% |
|  | Non-metastasis | 1 | 4 | 5 |  |
|  | Total | 5 | 6 | 11 |  |
| **Sphericity formula combined with CT** | Metastasis | 4 | 2 | 6 | 72.7% |
|  | Non-metastasis | 1 | 4 | 5 |  |
|  | Total | 5 | 6 | 11 |  |

Table S5 Statistical variability analysis for each of the two methods in post-neoadjuvant patients

|  | 2D formula | Sphericity formula | Decision tree model | Random forest model | B-ultrasound | CT | Sphericity formula combined with ultrasound | Sphericity formula combined with CT |
| --- | --- | --- | --- | --- | --- | --- | --- | --- |
| 2D formula | - | 0.5 | 0.33 | 0.33 | 0.5 | 0.665 | 0.33 | 0.33 |
| Sphericity formula 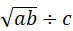 | 0.5 | - | 0.5 | 0.5 | 0.335 | 0.5 | 0.5 | 0.5 |
| Decision tree model | 0.33 | 0.5 | - | 0.682 | 0.193 | 0.33 | 0.682 | 0.682 |
| Random forest model | 0.33 | 0.5 | 0.682 | - | 0.193 | 0.33 | 0.682 | 0.682 |
| B-ultrasound | 0.5 | 0.335 | 0.193 | 0.193 | - | 0.5 | 0.193 | 0.193 |
| CT | 0.665 | 0.5 | 0.33 | 0.33 | 0.5 | - | 0.33 | 0.33 |
| Sphericity formula combined with ultrasound | 0.33 | 0.5 | 0.682 | 0.682 | 0.193 | 0.33 | - | 0.682 |
| Sphericity formula combined with CT | 0.33 | 0.5 | 0.644 | 0.682 | 0.193 | 0.33 | 0.682 | - |

A p value<0.05 was determined to be statistically significant.
